# Trans-labyrinthine Infra-trigeminal Approach for Recurrent Pontomedullary Cavernoma: A Step-wise Technical Note

**DOI:** 10.7759/cureus.5853

**Published:** 2019-10-07

**Authors:** Mayur Sharma, Jerry W Lin, Norberto Andaluz, Brian J Williams

**Affiliations:** 1 Neurological Surgery, University of Louisville School of Medicine, Louisville, USA; 2 Otolaryngology, University of Louisville School of Medicine, Louisville, USA

**Keywords:** translabyrinthine, recurrent, ponto-medullary, brainstem, cavernoma

## Abstract

Recurrent brainstem cavernoma is a challenging lesion due to the neurological risks associated with different surgical approaches. In this technical report, we present a 35-year-old female with a history of multiple brain cavernomas. She underwent midline suboccipital craniotomy and trans-fourth ventricle approach for resection of the brain stem cavernoma following two major bleeding episodes, one year prior to the presentation. Following the trans-labyrinthine infra-trigeminal approach, the patient recovered well postoperatively with a baseline neuro exam and was discharged to acute rehab on postoperative day 5 (POD5). The translabyrinthine approach is a safe and effective corridor for pontine or pontomedullary lesions in carefully selected patients. Appropriate selection of surgical approach (based on location), meticulous surgical technique, and intraoperative neuromonitoring help in maximizing surgical resection while minimizing neurological deficits.

## Introduction

Despite advances in micro-neurosurgical techniques, brainstem cavernoma (BC) continues to be a challenging lesion due to inherent risks associated with different surgical approaches [1-4]. Initial and recurrent hemorrhages from BC cause significant morbidity associated with the mass effect of the hematoma on critical and dense neural structures in the brainstem [5]. Recurrent hemorrhage from BC can be lethal if not managed by an experienced neurosurgeon at an experienced center [1-3]. Various surgical approaches, such as retrosigmoid, suboccipital, far lateral, infratentorial supracerebellar, orbitozygomatic, and translabyrinthine, have been described to approach these lesions with varying success [2,6-8]. There is a paucity of literature focusing on surgical approaches in patients who present with recurrent hemorrhage. In this technical report, we aim to provide a step-by-step description of a surgical approach (trans-labyrinthine infra-trigeminal) used in managing a complex patient who presented with recurrent BC hemorrhage.

## Technical report

Case illustration

**Figure 1:**
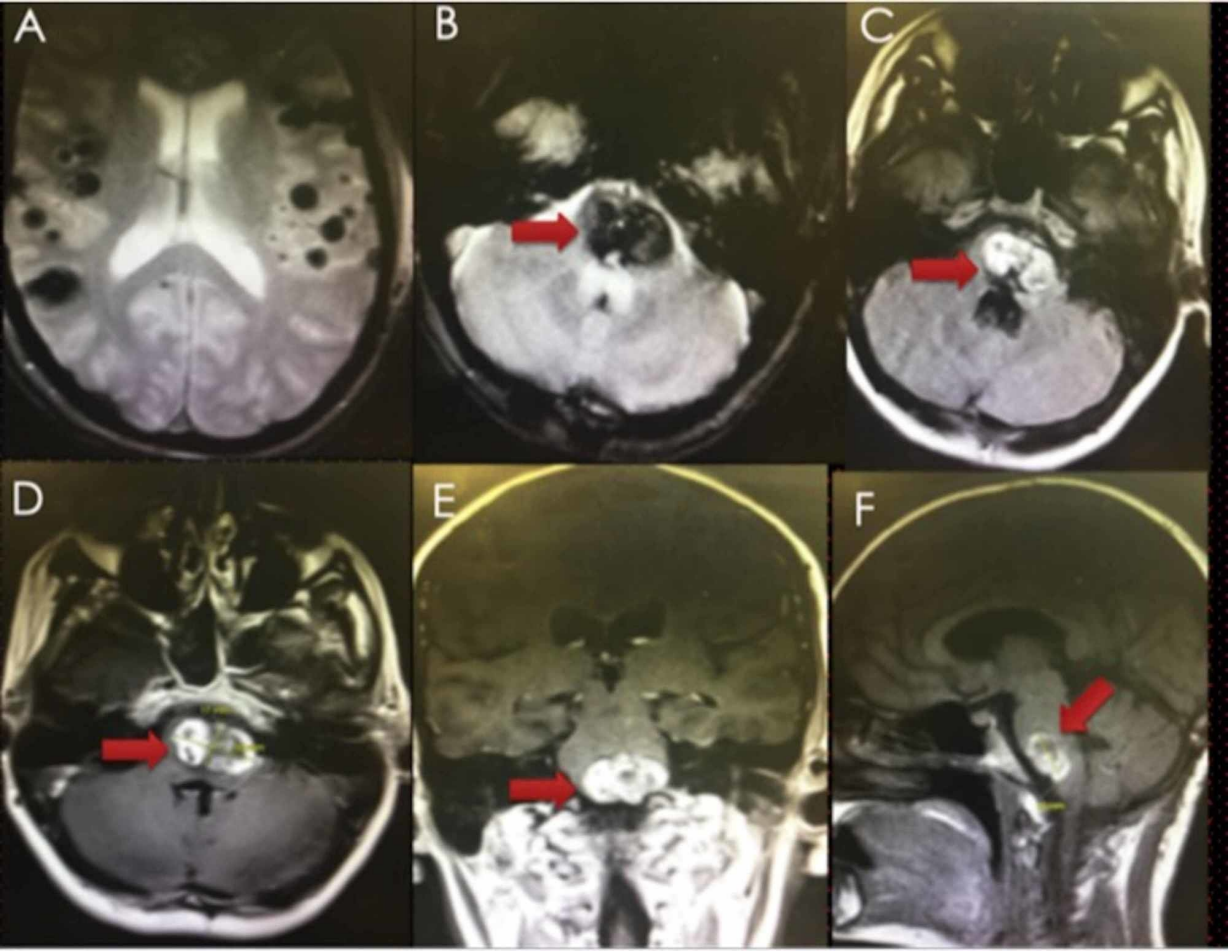
Preoperative MRI (GRE (A&B), T1 FLAIR (C), and T1W gadolinium contrast images (D-F)) showing multiple intracranial cavernomas (supratentorial A, pontomedullary B-F, red arrows). MRI: magnetic resonance imaging; FLAIR: fluid-attenuated inversion recovery

Our patient was a 35-year-old female with a history of multiple brain cavernomas. She had a significant past medical history of seizures controlled with Keppra and carbamazepine. She also has a history of smoking (0.5 pack for the last 10 years) and recreational drug abuse. She underwent midline suboccipital craniotomy and the trans-fourth ventricle approach for the resection of brain stem cavernoma following two major bleeding episodes, one year prior to the presentation. She recovered well; however, she developed major bleeding episodes two months and one month ago with worsening neurological symptoms from a residual cavernoma. She presented to us with bilateral abducens nerve palsy, left-sided facial paralysis (House Brackmann grade VI), left non-serviceable hearing loss, left-sided tongue weakness, and right hemiparesis (Grade 3/5 upper and lower limbs, Medical Research Council, UK) [9]. Magnetic resonance imaging (MRI) showed a contrast-enhancing intra-axial lesion involving the majority of the base of the pons and pontomedullary junction with multiple supratentorial cavernomas (Figure 1).

Technical pearls

Following the induction of general anesthesia, she was placed in the right lateral decubitus position with appropriate padding of all pressure points. A lumbar drain was inserted at the L4-5 interspace to facilitate brain retraction by draining cerebrospinal fluid (CSF) intraoperatively. She was then placed back in the supine position, a roll was placed under the left side, and the head was turned to the right. The head was fixed using 3-point Mayfield-Kees fixation and secured to the bed frame. Electrophysiological neuro-monitoring (motor evoked potentials, somatosensory evoked potentials, brainstem auditory evoked potentials, trigeminal, facial nerve electromyography, vagal, hypoglossal, and spinal accessory nerves monitoring) were applied. 

**Figure 2:**
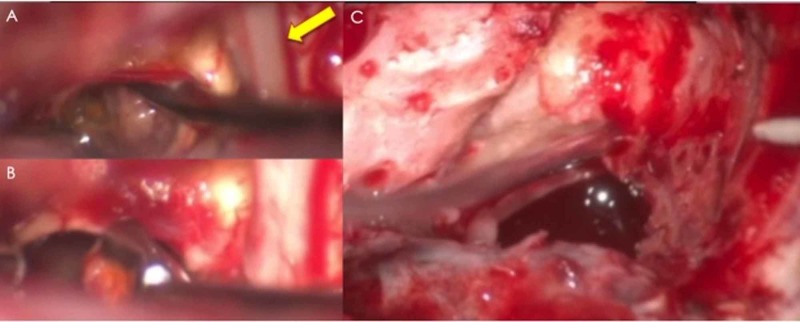
Intraoperative images showing the resection of a brain stem cavernoma using Rhoton’s dissector and pituitary ring curettes (A&B, yellow arrow points to cranial 7th/8th nerve complex). C shows the resection cavity with clear fluid by the end of the procedure.

Preoperative antibiotics were given and frameless neuronavigation was registered and verified. J.L. (ear, nose throat (ENT) colleague) then planned a curvilinear incision behind the ear in order to expose the transmastoid translabyrinthine approach to the brainstem. Following mastoidectomy, the dura of the middle fossa floor was exposed. Superior petrosal and sigmoid sinuses were exposed as boundaries of Trautmann’s triangle. The posterior semicircular canal was drilled along the medial border of the incision to expose the cerebellopontine angle region with pons and medulla. Given the location of the lesion, frameless neuronavigation was used to confirm our entry site into the brainstem. An arachnoid knife was used to dissect through the arachnoid of the cerebellopontine angle cistern and identify the area of the brainstem. An operating microscope was then used to perform microdissection and the arachnoid knife was used to incise the surface of the brainstem and the pia-mater. Bayonet forceps were then used to dilate the aperture into the brainstem and identify the frank hematoma cavity, which was encountered after dissecting approximately 5 mm into the brainstem. Series of micro-dissectors (Rhotons #1, #2, and #3 ) and pituitary ring curettes were then used to excise the cavernous malformation in a piecemeal fashion. Multiple vessels within the cavity were coagulated to obtain hemostasis. After satisfactory resection, hemostasis was obtained with a small amount of FloSeal (Baxter International, IL, US​​​​​​) and bipolar cautery. Abdominal fat and temporalis fascia were used to reconstruct the mastoid antrum and DuraGen® (Integra, Plainsboro, NJ, US) was used to cover the dural defect. The incision was closed in layers, and a mastoid wrap was applied. There were no changes to neuromonitoring throughout the case, with a slight improvement in motor evoked potentials on the right side. The patient was extubated and transferred to the intensive care unit in stable condition (Video 1 and Figure 2).

**Video 1:** In this video, we reviewed the trans-labyrinthine infra-trigeminal surgical approach with pertinent details related to microneurosurgical resection of a recurrent brainstem cavernoma.

**Figure 3:**
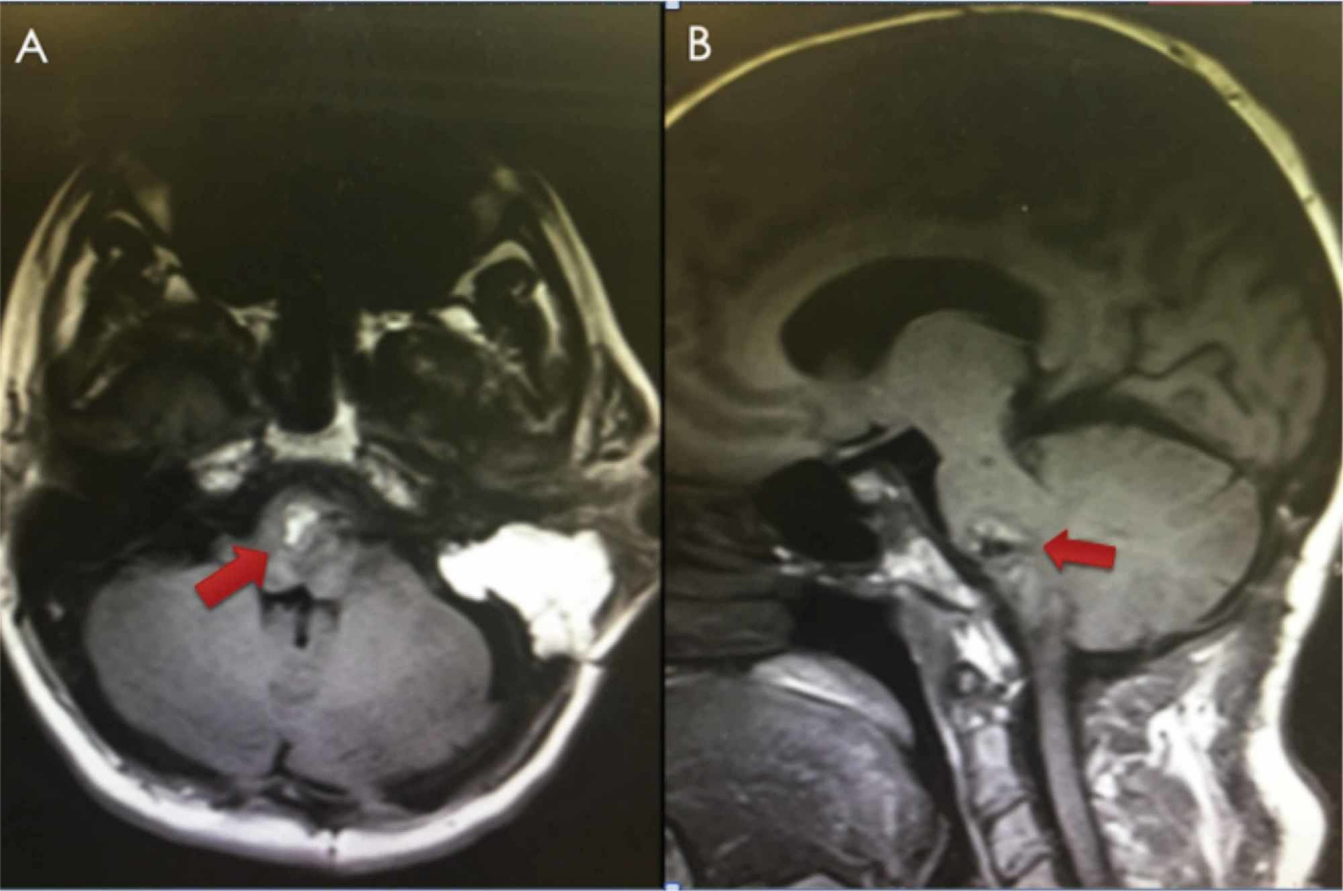
Postoperative MRI (T1W without contrast) showing satisfactory resection of the pontomedullary lesion with a fat graft in the resection cavity (red arrows). MRI: magnetic resonance imaging

**Figure 4:**
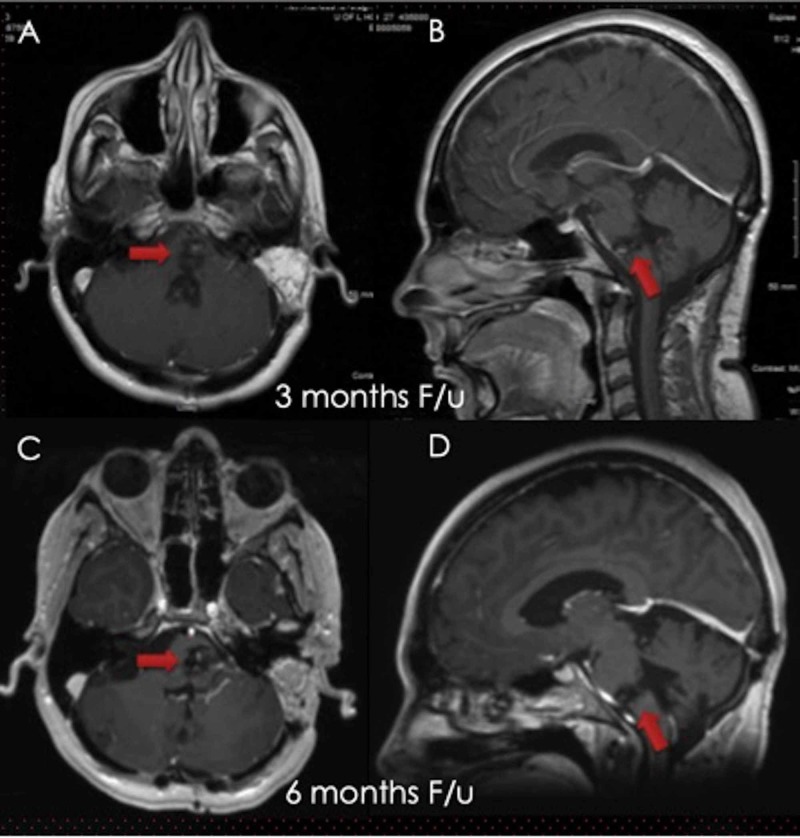
Follow-up MRI (T1W with contrast) at three months (A&B) and six months (C&D) showed a stable size of the resection cavity of the pontomedullary lesion with no recurrence or repeat hemorrhage (red arrows). MRI: magnetic resonance imaging

The patient recovered well postoperatively with a baseline neuro exam (no change in the cranial and motor exam) and was discharged to acute rehab on POD5. Postoperative MRI showed satisfactory resection of the pontomedullary mass (Figure 3). At the three and six-month follow-ups, MRI showed the stable appearance of the lesion with a gradual improvement in abducens nerve function (Figure 4).

## Discussion

Multiple cavernous malformation (CM) occur in approximately 19% of adult patients with CM [10] and approximately 30% of pediatric patients with CM [11]. BC accounts for approximately 18% of all intracranial cavernomas [10]. The annual hemorrhage rate of all CM is reported as 2.5% (1.3%-5.1%) per patient-year at 5081.2 patient-years follow-up [10,12]. A recent meta-analysis reported an annual hemorrhage and re-hemorrhage rate of 0.3% and 6.3%, respectively, for non-BC as compared to 2.8% and 32.3% for BC [5]. Most of the re-hemorrhages have been reported to occur within two years of initial hemorrhage with a median time of 10.5 months [5]. Similarly, our patient presented within one year of the initial hemorrhage with repeat episodes of hemorrhage. Also, the morbidity associated with hemorrhages (at all locations) is quite significant, with only 40% of patients having been reported to be completely normal and 80% with minimal deficits, with mortality of 2.2% [5]. The natural course of recurrent BC is likely to be lethal without intervention [1]. Early intervention (<6 weeks) has been shown to improve clinical outcome scores in a series of 397 patients with 52% of patients showing complete resolution of preoperative symptoms and a new permanent postoperative deficit was noted in 32% of patients [3]. Stereotactic radiosurgery (SRS) has been shown to reduce the annual hemorrhage rates in patients with BC from 25%-40% (pre-Gamma knife) to 2.4% -3.9% at two years and 1.48% at five years after SRS [13-15]. However, the risk of an annual hemorrhage tends to increase after five years to 4.6%. Given this high rate of symptomatic hemorrhage/re-hemorrhages in patients with BC along with the morbidity associated with surgical intervention, it is critical to consider treatment options in these patients after discussing the risks and benefits of individual treatment modality.

Surgical approaches are potentially guided by the location of the lesion within the brainstem [2,6-8,16]. Abla et al. [2] have recommended the retro-sigmoid, sub-occipital±telovelar, or retro-sigmoid/lateral supra cerebellar infra-tentorial approaches for a pontine cavernoma and the retro-sigmoid or far-lateral approach for cavernoma located at the pontomedullary junction. Invasive approaches, such as anterior petrosectomy, trans-cochlear, and full orbito-zygomatic craniotomy can be considered in patients with recurrent BS cavernomas in difficult-to-access locations. We believe that there may be a small residual cavernoma during the initial suboccipital approach (due to the lateral extent of the cavernoma at the pontomedullary junction) that may have lead to a recurrence. In view of the location of the recurrent lesion and left-sided cranial nerve deficits, including hearing loss, we decided to choose the left trans-labyrinthine approach with intraoperative monitoring. The main advantage of this surgical approach, as compared to other approaches (such as retro-sigmoid or retro-labyrinthine pre-sigmoid corridors), includes a shorter surgical path and likelihood to achieve gross total resection. However, complete hearing loss is the main disadvantage of using this corridor (our patient had non-serviceable hearing loss). Our report provides insight into the technical nuances and advantages offered by this approach in a patient with recurrent hemorrhage from BC with a favorable outcome.

## Conclusions

The translabyrinthine approach is a safe and effective corridor for pontine or pontomedullary lesions in carefully selected patients. Appropriate selection of surgical approach (based on location), meticulous surgical technique, and intraoperative neuromonitoring help in maximizing surgical resection while minimizing neurological deficits.
